# Toward lower-emission beef production: research gaps and opportunities

**DOI:** 10.1093/af/vfag046

**Published:** 2026-09-10

**Authors:** Logan R Thompson, Sara E Place, Troy N Rowan

**Affiliations:** Department of Animal Science and Industry, Kansas State University, Manhattan, KS 66506; AgNext, Department of Animal Sciences, Colorado State University, Fort Collins, CO 80523; Department of Animal Science, University of Tennessee, Knoxville, TN 37996

**Keywords:** beef production, climate neutrality, GHG mitigation, producer viability

Implications

- Cattle production in the United States utilizes 35% of the country’s land mass and is economically critical for more than 600,000 beef producers, however mitigating methane produced via enteric fermentation remains a challenge in nonconfinement production systems.
- Cattle producer organizations have set industry-wide targets to improve the sustainability of the industry, which include a reduction in Air and Greenhouse Gas Emissions to achieve climate neutrality by the year 2040.
- While the industry has reduced emissions per kg of beef produced by more than 40% over the last half-century, further advancements are needed to achieve the industry’s goal of climate neutrality.
- No “silver bullet” exists to reduce emissions. Rather, advancements in nutrition, management, genetics, quantification, and so forth will be required.

## Background

In recent years, there have been significant public and policy commitments to reduce greenhouse gas (GHG) emissions from all industry sectors. This has been driven by an increasing concern over the changing climate and the role that anthropogenic emissions play in this process. The agricultural sector has not been immune to this conversation, and some reports estimate that food production may account for up to 34% of total global emissions (Crippa et al., 2021). Within the beef cattle industry, the predominant GHG is enteric methane (CH_4_), which is a byproduct of ruminal fermentation. The majority (approximately 85%) result from grazing systems within the cow-calf and stocker sectors of the beef industry (Rotz et al., 2019). When including all GHG produced during the lifecycle of beef production, approximately 80% of emissions arise from these sectors, indicating that we must focus our efforts on grazing systems, and in particular on enteric CH_4_ emissions (Rotz et al., 2019). Further, the rise of NetZero commitments made within the US beef supply chain means that research efforts need to be refocused on developing climate-smart solutions for grazing sectors. However, grazing sectors present unique challenges for the research community and industry at large. These challenges are related to significant variation in production systems both within and across regions; significant knowledge gaps (gaps that do not exist with confinement systems), such as measuring feed intake; and a lack of funding for testing mitigation strategies in grazing sectors. Feedlots and other confinement production systems still are in need of investment; however, achieving meaningful GHG emission reductions across the suite of emission types will require all sectors to make progress. Further, production systems have differing portfolios of emissions, thereby requiring actions specific to the reduction of specific emission types, from varying pathways, without increasing the production of other GHG types. In confinement, the production of reactive nitrogen (N) species and the reliance on cereal grains have caused issues with N contamination in other ecosystems and concerns about soil health and carbon (C) stocks in crop grounds, to name a couple of issues (Thompson et al., 2025). In this paper, we will discuss the outcomes of the US Roundtable for Sustainable Beef (USRSB) roadmap development process, as it relates to air and GHG emissions from the beef production supply chain. Key areas of improvement and future opportunities highlighted during this process include: (1) Measurement and Modeling of Air and GHG Emissions, (2) Grazing Sector Reductions, (3) Feedlot Sector Reductions, and (4) Maintaining Producer Viability (Figure 1).

**Figure 1.**
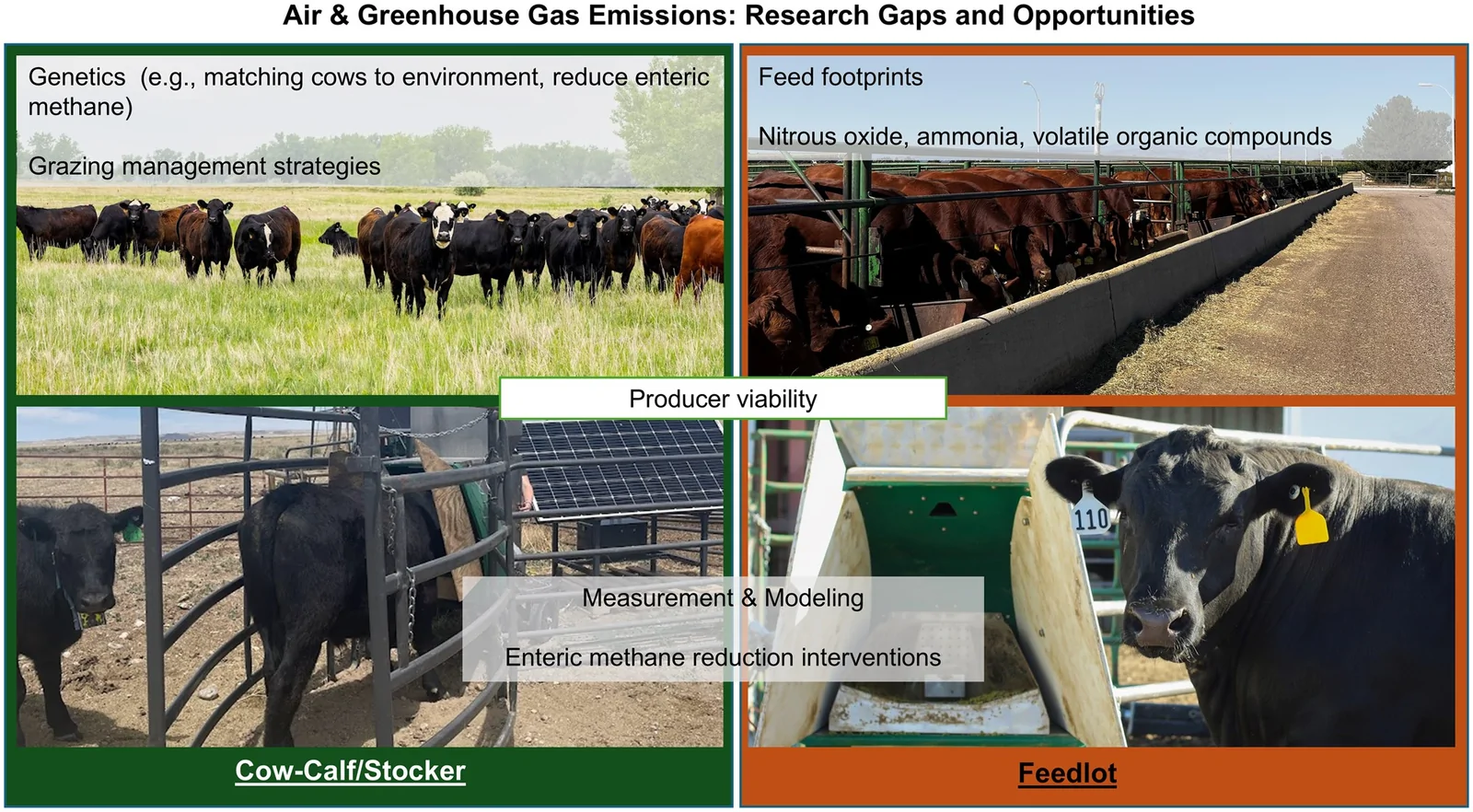
Approaches to reducing air and greenhouse gas emissions across cow-calf, stocker, and feedlot sectors.

## Research gaps and areas of opportunity

### Measurement and modeling of air and GHG emissions

Core to mitigation of emissions from beef cattle production is accurate and precise measurement methods to assess baseline emissions, test and confirm mitigation strategies, and provide the best available empirical evidence to create, evaluate, and update models. Modeling plays a critical role as measurement of emissions is not possible in all contexts, and modeling can improve the understanding of the system impacts of mitigation strategies. Measurement, reporting, and verification (MRV) approaches required by carbon registries, corporate reporting, and industry goals tracking typically require either measurement, modeling, or a combination of the two. However, standardization of methodologies for quantifying air and greenhouse gas emissions is largely lacking. Measurement of emissions, whether enteric CH_4_ from cattle or emissions from manure or pen surfaces, is often costly, complex, and typically confined to university or research institute settings. Expanding the ability to measure emissions in commercial production conditions or determine better proxies for emissions would accelerate progress in both baselining and mitigation (Tedeschi et al., 2022).

To evaluate and improve models of emissions, empirical observations of emissions are needed. A key opportunity is pooling data across research groups and universities to create larger independent datasets with observations across cattle types, climates, and production environments. However, building national or regional data sharing consortiums faces barriers with regard to data interoperability, data ownership, and funding for maintaining a sustainable data repository. Concomitant with any data sharing consortium would be creating common standards for emissions measurement and a common list of data collected across all sites (e.g., feed nutrient composition, feed intake, body weight gain, enteric methane) to increase the utility of the data for modelers (McNamara et al., 2016).

### Grazing sector reductions—role of management and genetics

Given the outsized impact that the grazing sector contributes to overall GHG emissions from the beef supply chain (approximately 85% between cow-calf and stocker sectors), considerable research is still needed to develop sector-specific mitigation strategies. Additionally, as highlighted above with respect to where research occurs, the ability to replicate commercial grazing operations in controlled research environments is a challenge in the creation of robust mitigation strategies. While the direct supplementation of enteric CH_4_ inhibitors such as 3-Nitroxypropanol (3-NOP) may be viable in the future years, there are existing low-cost strategies that may be incorporated by improving landscape and animal management and animal selection (DeRamus et al., 2003; Thompson and Rowntree, 2020; Gandolfo et al., 2025).

Compared with confinement, grazing systems make the delivery of management or feed supplement interventions challenging. Access to cows is irregular, especially in more extensive production environments in the Western US. This makes genetic selection a promising and practical approach to decreasing enteric emissions. Enteric CH_4_ emission is a moderately heritable, complex trait with estimates ranging from 0.15 to 0.40 (Dressler et al., 2024). This suggests that sufficient additive genetic variability exists within populations to make genetic progress. Whereas feed supplements must be continually delivered to animals, the changes produced by genetic selection are both permanent and cumulative, meaning that progress made by the present generations is not lost in subsequent ones.

Breeding programs must account for the full range of profit drivers, and as such, novel traits like enteric CH_4_ must be balanced with others of economic importance (e.g., weaning weight, cow longevity, carcass merit). Genetic correlations indicate the degree to which a common set of genes affects multiple traits in the same direction. Raw CH_4_ emission is highly genetically correlated with feed intake (*r*_G_ = 0.51) and moderately genetically correlated with other growth and end-product traits (*r*_G_ Metabolic Weight = 0.27) (Crowley et al., 2026). These relationships point to a necessary evaluation of enteric emissions relative to feed intake (CH_4_ yield) or product output (CH_4_ intensity) (Figure 2). From the perspective of supply chains interested in reducing the GHG footprints, reductions in CH_4_ intensity driven by productivity increases offer an encouraging approach. These reductions in per-unit CH_4_ emission are likely to accompany overall increases in production efficiency related to feed conversion, fertility, and animal health. In other contexts, direct inclusion of CH_4_ reductions into the breeding objective may be necessary, particularly in countries where governments are incentivizing absolute emissions reductions (e.g., New Zealand or Ireland). Long-term selection experiments for CH_4_ emissions in a New Zealand sheep population suggest that selection does not negatively affect maternal traits, health outcomes, or carcass quality (Hickey et al., 2022; Rowe et al., 2022).

**Figure 2.**
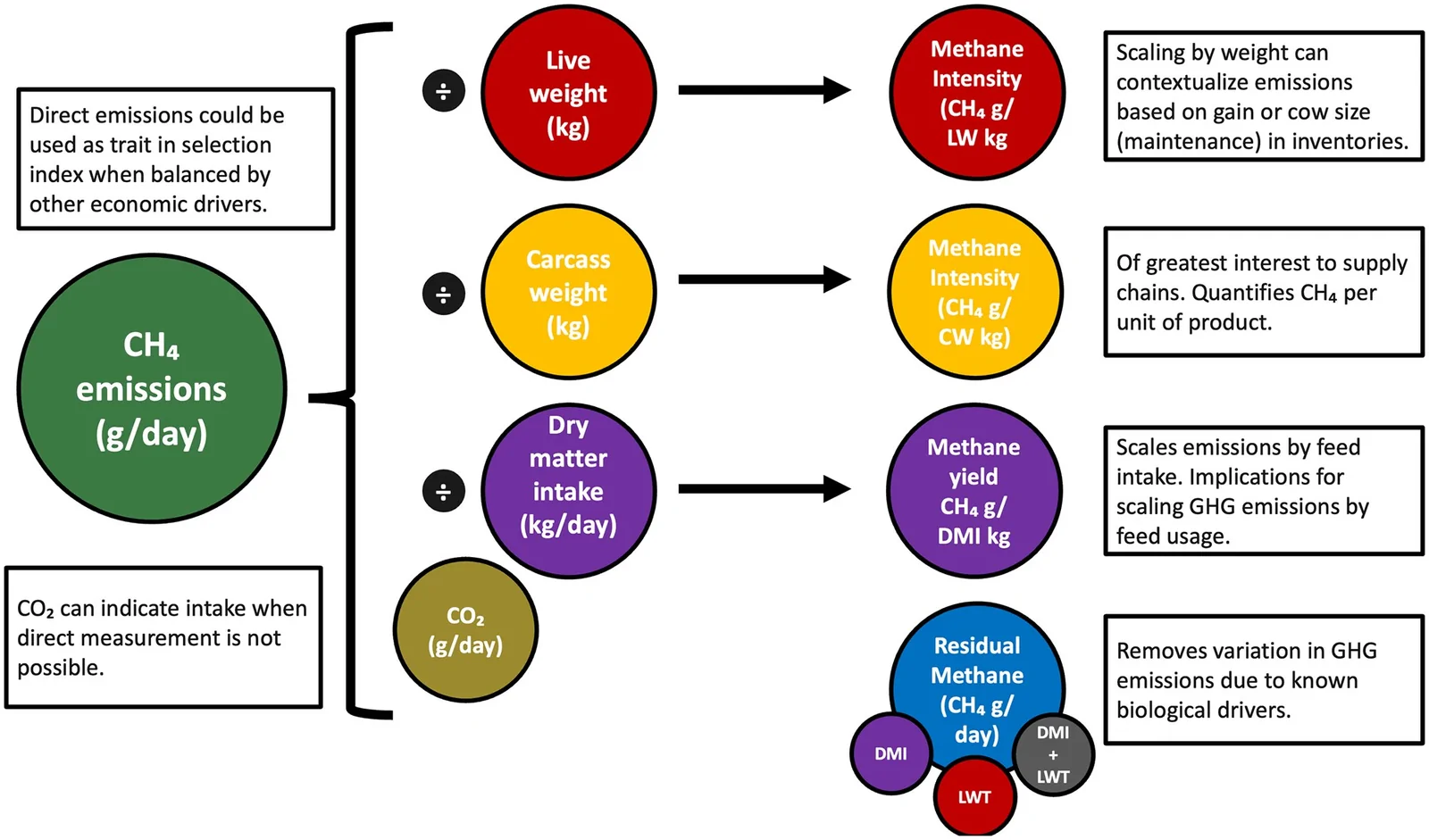
Definitions of methane emission-related traits and their potential applications to genetic evaluation and GHG accounting schemes.

The development of genetic selection tools (i.e., expected progeny differences—EPDs) requires the collection of thousands of phenotypes on animals within a population or breed (Dekkers et al., 2021). Routine phenotype collection remains the greatest hurdle to developing EPDs for emissions traits. Current methods for measuring individual animal emissions remain expensive and difficult to deploy in grazing settings. Respiration chambers remain the gold standard for measuring emissions, but are incapable of capturing the scale of records needed for genetic evaluations and removing cattle out of their grazing context. GreenFeed emissions monitoring systems (C-Lock Inc., Rapid City, SD) have been widely deployed as research tools and can measure higher volumes of animals in grazing settings (Hristov et al., 2015). Despite improved throughput compared with respiration chambers or other methods like SF_6_ tracer techniques, GreenFeed gas flux phenotypes remain cost-prohibitive for unsubsidized collection on producer farms. Methane and carbon dioxide sniffers have been successfully deployed in dairy settings for routine on-farm emissions recording. These devices are substantially cheaper and easier to run, but measure only gas concentration, rather than flux like the GreenFeed, resulting in less accurate measurements. While this may reduce their utility for characterizing absolute differences between scientific treatments, they are still useful in genetic phenotyping initiatives aimed at correctly ranking animals. Comparisons in dairy contexts have demonstrated moderate phenotypic correlations and high genetic correlations between sniffers and GreenFeed systems (van Breukelen et al., 2023). Ongoing developments are adapting sniffers to fit into beef production settings, like in-pasture weigh systems or feed intake bunks.

To balance all traits of economic importance, breeding programs utilize selection indexes that weight traits by their economic importance to a larger breeding objective (Hazel et al., 1994). Indexes rely on clear economic values for traits to be included. This may be pricing data for revenue-generating traits like weaning or carcass weight, or cost data for input indicator traits like fertility or milk production. This means that selection on CH_4_-related traits will not occur in a vacuum, but alongside dozens of other drivers of efficiency and profitability across the beef system. At present, no market signals exist to add CH_4_ reductions into the breeding objective, but inclusion could occur when clearer signals exist or through a desired gains approach. Given the positive genetic correlations between CH_4_ emission, feed intake, and animal growth potential, continued selection with no pressure on enteric emissions would result in higher per-animal gross emissions, but likely decrease emissions intensity (Richardson et al., 2023).

Outside of genetic selection, and given the current limited viability of direct CH_4_ inhibitors, managing the forage base and supplementation program to maximize the nutritional status of grazing animals is a promising avenue to reduce emissions (Thompson and Rowntree, 2020). Starting with the forage base, management strategies that improve forage quality throughout the grazing season can reduce emissions per unit of intake by reducing dietary fiber concentration and improving digestibility. This can occur through both grazing management strategies (such as rotational grazing) and changing the forage base to include more high-quality forages. This can be advantageous for both the parental populations and growing animals to improve animal performance. However, drastic changes to a forage base are not always viable given geographic or producer limitations. Long-standing best management practices, often referred to in the United States as BMPs, still hold value in reducing grazing-based emissions through improvements in nutritional status and animal performance (DeRamus et al., 2003; Thompson and Rowntree, 2020). Common supplementation programs that improve the health of reticulo-rumen fermentation, such as protein supplementation during forage dormancy or energy supplementation when dietary fiber is deficient, serve to balance nutritional needs within the rumen. These programs were developed, initially, to improve the performance of mature cows and growing calves, but concurrently result in reduced emissions per unit of gain or product (Thompson and Rowntree, 2020). Furthermore, matching management to the environment (such as timing and duration of grazing) has been estimated to reduce emissions per unit of land and maximize landscape productivity (Bernardi et al., 2025). These management strategies are not lacking in scientific merit, but do require continued outreach to expand adoption across the industry within appropriate regional applications.

### Feedlot sector reductions—carbonaceous and nitrogenous emissions

The feedlot sector plays a key role in the US beef industry, contributing approximately 50% of the liveweight gain of cattle, while only contributing approximately 15% of the sector’s total GHG emissions (Rotz et al., 2019; Betts and Harborth, 2024). In US feedlots, cattle are typically fed grain-based diets including technologies and feedstuffs that can lower enteric methane emissions (e.g., ionophores, supplemental fat), and consequently, the enteric CH_4_ emissions from the feedlot phase are lower per unit of feed intake compared to the grazing phases of the industry (Johnson and Johnson, 1995). However, opportunities remain to investigate cost-effective and safe strategies to reduce enteric CH_4_ emissions from feedlot cattle, including feed additives such as 3-nitrooxypropanol (3-NOP), bromoform-containing additives, and natural products (e.g., *Asparagopsis taxiformis*). An important area of research for feed additives or any other CH_4_ mitigation strategy is better understanding the impacts on rumen fermentation and productivity. While it is often cited that enteric CH_4_ reductions can increase feed energy available for productive functions, empirical evidence is mixed (Morgavi et al., 2023). A better understanding if CH_4_ reduction can translate into feed efficiency benefits or changes in days on feed could help adoption, as mitigation strategies with neutral productivity impacts may be hard to adopt without substantial financial incentives for cattle producers (Luke and Tonsor, 2024).

Beyond enteric methane emissions, emissions associated with feed production and nitrous oxide (N_2_O) emissions from manure (from pen surface or when applied to land) account for substantial amounts of additional feedlot-level emissions (Rotz et al., 2019). Many feedlots purchase significant portions of the total feedstuffs used, making the origin of the feeds and their associated GHG emissions unclear. Advances in feed GHG footprinting methods and tracking could better link reductions in feed production emissions to beef cattle production. Additionally, improvements in feed efficiency may translate into reductions in the feed GHG footprint of feedlot cattle production. Nitrous oxide emissions from feedlot pen surfaces can be highly variable both spatially and temporally (Waldrip et al., 2016). These emissions are difficult to quantify from commercial pen conditions, as measurement techniques like flux chambers require physically placing a measurement apparatus in a pen with cattle, leading to potential cattle interaction with the measurement equipment. Other measurement strategies, such as micrometeorological techniques, measure multiple pens or whole facility-scale emissions, allowing for assessment of emissions in commercially relevant conditions but making replicated experiments difficult. Mitigation of N_2_O emissions per unit of live weight gain can be achieved through increased productivity and use of production technologies that increase N retention (Stackhouse-Lawson et al., 2013; Waldrip et al., 2016). There may be potential for pen surface amendments to reduce N_2_O (and ammonia; NH_3_), such as urease inhibitors; however, research on amendments is still relatively scarce, and practical, cost-effective solutions for cattle feedlots are lacking.

Beyond GHG, NH_3_ emissions and volatile organic compounds (VOCs) are other emissions from cattle feedlots that may negatively impact air quality, cause ecosystem impacts, and lead to unwanted odors. Similar to N_2_O, NH_3_ emissions can be difficult to measure from cattle feedlots and originate from the pen surface and manure rather than the cattle themselves. Ammonia losses can be significant, representing up to 50% of the N consumed by finishing cattle, potentially lost as NH_3_ in feedlots depending upon diets, management, and climatic conditions (Lee et al., 2025). Precision feeding techniques, technologies that increase N retention, and pen surface amendments that lower pH or disrupt N cycling processes can reduce NH_3_ emissions from cattle feedlots. However, similar to N_2_O emissions, viable pen surface amendments are lacking for commercial cattle feeders. Odors from feedlots can be caused by both NH_3_ and VOCs (Parker et al., 2007). Research characterizing VOC emissions and interventions to mitigate their emissions is fairly limited; however, it is understood that the moisture conditions of pens, dietary ingredients, and pen management may influence VOC emissions (Woodbury et al., 2015).

### Producer viability

Producer buy-in and trust are critical aspect that has to be considered when developing emission mitigation strategies, regardless of production sector. Filtering out strategies that lack this practical attribute is critical to the success of any practice development and incentivizing behavior change (Figure 3). For example, a technology that has been available to beef producers for numerous years and has lacked adoption is a commercial vaccine that effectively reduces E. coli shedding by more than 50% (Tonsor and Schroeder, 2015). While the tool holds scientific merit, producers lack the market signal to incorporate its use into their management plan, and as such, it is not practical for them to adopt without incurring losses (Tonsor and Schroeder, 2015). Research occurring in a vacuum will not result in the desired improvements by stakeholders. The development of a continuous feedback loop between researchers and producers will aid the pace of progress.

**Figure 3.**
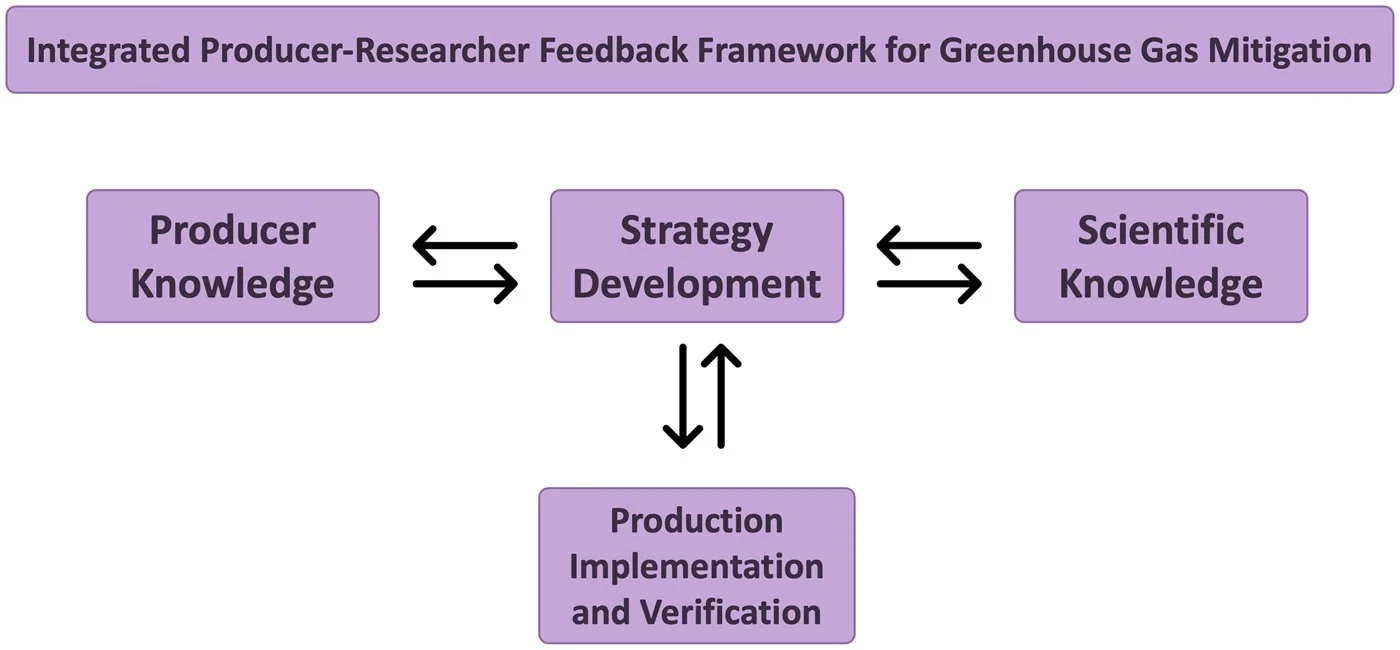
An integrated producer-researcher feedback framework for greenhouse gas mitigation.

Unlike other more integrated livestock industries, beef production relies on thousands of independent cow-calf operations to make individual genetic selection decisions. While CH_4_ emissions can be reduced via genetics, selection decisions ultimately rest on the shoulders of producers. In the absence of concrete market signals for reducing CH_4_ emissions, continued progress on traits that improve overall system efficiency (e.g., improved fertility, reduced cow maintenance needs, improved feed efficiency, fewer days on feed) will be the primary path toward improved CH_4_ intensity of beef production. These traits are already largely part of the industry’s larger breeding objective. Consequently, even in the absence of explicit selection on enteric emissions, the industry has improved emissions intensity through its continued selection on growth-related traits, and more recently on cow productivity and feed efficiency (Capper, 2011; Crawford et al., 2022). Continued increases in production efficiency, especially in beef systems in the Global South, will continue to reduce emissions intensities globally. This progress will rely on sustained engagement with producers through extension and technical education regarding best production practices, especially those with impacts on sustainability outcomes (e.g., crossbreeding, balancing output and cow energy needs).

## Conclusions

Reducing GHG emissions from the beef supply chain will require a coordinated effort across research groups and between researchers and producers. Due to the majority of emissions arising from grazing sectors, in order for meaningful reductions to occur, an increased focus and investment are needed to develop practical strategies that are regionally applicable and easy to adopt. Genetic selection, improved forage and animal management, and continued advancements in overall production efficiency represent some of the most immediately actionable pathways to reduce emissions in grazing systems. Concurrently, continued advancements within the feedlot sector focused on improvements in efficiency, methane inhibitors, manure management, and feed sourcing are essential, given the shift in the emission portfolio for that sector.

This scientific progress cannot happen in isolation, as technical feasibility is not sufficient to ensure widespread adoption. Working with producers to ensure viability and working within existing market signals is critical during the scientific process. This process will also require investments to improve measurement infrastructure and modeling capabilities to improve the accuracy of emission estimates and reductions in commercial operations. Lastly, improving coordination and collaboration across research groups will require overcoming numerous barriers, but will aid the pace of progress and develop more robust mitigation strategies.

## Author Biographies

**Dr. Logan Thompson** is an Assistant Professor at Kansas State University and Director of Rannells Ranch Flint Hills Prairie Preserve. A native Texan, Logan got his bachelor’s degree at Texas Tech prior to stops at Oklahoma State and Michigan State University. Logan’s research and extension program at K-State examines grazing management and rangeland ecology in the Flint Hills.

**Dr. Sara Place** is an Associate Professor and expert in livestock systems sustainability with over a decade of experience in academia, industry associations, and private industry. She joined Colorado State University’s AgNext, a research collaborative focused on sustainable animal agriculture, in August 2022. Sara’s research focuses on enteric methane emissions measurement and mitigation from cattle. Prior to CSU AgNext, she was the chief sustainability officer for Elanco Animal Health, senior director for sustainable beef production research at the National Cattlemen’s Beef Association, and an assistant professor in sustainable beef cattle systems at Oklahoma State University. She received her PhD in Animal Biology from the University of California, Davis, and a BS in Animal Science from Cornell University. Sara is a native of upstate NY, where she grew up on a dairy farm.

**Dr. Troy Rowan** is an Associate Professor in the Department of Animal Science at the University of Tennessee, where his research program focuses on the application of genomic and quantitative genetic tools to improve the efficiency, sustainability, and profitability of beef cattle production systems. His work uses large-scale genomic datasets and novel phenotypes to identify the genetics underlying grazing cow efficiency, adaptation, and resilience. His group’s research spans the continuum from fundamental biological discovery to applied genomic selection strategies, all with the goal of developing actionable tools for seedstock producers, breed associations, and commercial cow-calf operations. In addition to his research, Dr. Rowan is engaged in extension and outreach efforts aimed at communicating the value of genomic technologies to beef producers around the globe. Troy’s extension work supports the Tennessee Master Beef Producer Program and other national organizations (the Beef Improvement Federation, NCBA, and US Roundtable for Sustainable Beef).
